# Effects of Replacing Fishmeal and Soybean Protein Concentrate with Degossypolized Cottonseed Protein in Diets on Growth Performance, Nutrient Digestibility, Intestinal Morphology, Cecum Microbiome and Fermentation of Weaned Piglets

**DOI:** 10.3390/ani12131667

**Published:** 2022-06-28

**Authors:** Li Wang, Wenjun Gao, Huangwei Shi, Qile Hu, Changhua Lai

**Affiliations:** State Key Laboratory of Animal Nutrition, College of Animal Science and Technology, China Agricultural University, Beijing 100193, China; q15928806587@163.com (L.W.); wjgao1119@163.com (W.G.); shw1161402802@163.com (H.S.); huqile44@163.com (Q.H.)

**Keywords:** cecum microbiota, degossypolized cottonseed protein, weaned piglets

## Abstract

**Simple Summary:**

Highly digestible and high-quality proteins are especially needed in weaned pigs to alleviate weaning stress in piglets. Fishmeal (FM) and soybean protein (SPC) are two commonly used protein supplements in the diets of weaned pigs, but the high prices of those two kinds of ingredients have prompted a search for an alternative cost-effective protein source. After the removal of anti-nutritional factors, degossypolized cottonseed protein (DCP) shows potential as an alternative to FM and SPC. In this study, the effects on weaned piglets of replacing FM and SPC with DCP in diets were evaluated. The results showed that replacing FM with DCP weakens the small intestinal morphology and decreases nutrient digestibility, but improves the community structures of cecum microbiota that relieve these negative effects without impairing the growth performance of weaned piglets.

**Abstract:**

The inclusion of high-quality proteins is commonly used in swine production, especially in weaned pigs. Our research investigated the effects of replacing fishmeal (FM) and soybean protein concentrate (SPC) with degossypolized cottonseed protein (DCP) on the growth performance, nutrient digestibility, intestinal morphology, cecum microbiota and fermentation in weaned pigs. A total of 90 pigs were used in a 4-week trial. Pigs were randomly assigned to three dietary treatments (initial BW 8.06 ± 0.26 kg; six pigs per pen; five pens per treatment), including a basal diet group (CON) with a 6% SPC and 6% FM; two experimental diets group (SPCr and FMr) were formulated by replacing SPC or FM with 6% DCP, respectively. There were no differences in growth performance and diarrhea rate among three treatments except for the ADFI during day 14 to day 28. Using the DCP to replace FM would weaken the jejunum morphology and decrease the nutrient digestibility of pigs during day 0 to day 14. However, replacing FM with DCP can improve the community structure of cecum microbiota, and may relieve these negative effects. In conclusion, DCP can be used as a cost-effective alternative protein supplement.

## 1. Introduction

In the weaning period, piglets undergo a transition from a highly digestible liquid milk to a solid diet including complex protein [1]. Under this circumstance, the drastic changes to the food composition present challenges to the fragile intestinal tract of weaned piglets. Highly digestible and high-quality proteins are especially needed in weaned pigs to alleviate weaning stress and supply nutrients for piglets. Fishmeal (FM) and soybean protein concentrate (SPC) are two commonly added protein supplies for weaned pigs due to their high contents of essential amino acids, and can be fed to piglets without adversely affecting performance [2,3]. However, two problems remain. First, the price of soybean has risen in recent years due to competition between human consumption and the livestock industry, especially in the context of the significant growth of pig stock in China in recent years. Second, high demand and limited resources have caused the price of FM to be high, and the potential risk of animal protein also limits its use [4]. Therefore, the possibility of replacing SPC and FM with alternative protein sources could reduce economic costs and contribute to more sustainable pork production.

Cotton is cultivated in large quantities in China, with a production of 6.42 million tons in 2021 [5]. This kind of crop is mainly planted for its fiber, but more cottonseed is produced than the lint fiber ginned from cotton. After extracting oil from cottonseed, cottonseed meal is frequently used in animal feed and garden fertilizers due to its high protein content. Cottonseed proteins are sequentially extracted from defatted cottonseed meal. Even though cottonseed meal and cottonseed protein have high nutritional value due to their high protein contents, the output of free gossypol limits its use in pig farming [6]. Gossypol may be either free or bound. Bound gossypol is not toxic to pigs, but free gossypol is toxic [7]. Degossypolized cottonseed protein (DCP) is produced by the solvent extraction of water-soluble carbohydrates and free gossypol from cottonseed meal, which finally contains high-quality protein and a low concentration of free gossypol [8]. Compared with SPC and FM, the lower price and similar high-quality protein make DCP a potential protein source for weaned pigs.

However, limited information is available on the effects on pigs’ intestinal morphology, cecum microbiota and fermentation of replacing SPC and FM with DCP. Therefore, the objective of this study is to evaluate the effects on weaned pigs’ growth performance, nutrient digestibility, intestinal morphology, cecum microbiota and fermentation of replacing SPC and FM with DCP.

## 2. Materials and Methods

All procedures used in this experiment were approved by the China Agricultural University Institutional Animal Care and Use Committee (Beijing, China). All animal trials were conducted in the China Agricultural University Animal Experimental Base (Fengning, China). In this study, the DCP was kindly provided by Sino-leader Biotech Co., Ltd. (Beijing, China). The chemical compositions of DCP, FM and SPC were analyzed and are presented in Table 1.

### 2.1. Animals, Diets and Experimental Design

A total of 90 healthy 28 day aged crossbred pigs [Duroc × (Landrace × Large White)] with an initial BW of 8.06 ± 0.26 kg were used in this study. They were both from 10 sows (average BW = 253 kg, parity = 3) in the same batch and the lactation period lasted 21 days. After 7 days of weaning adaption period, they were assigned to 3 dietary treatments in a completely randomized design. Each treatment diet was fed to 5 replicated pens with 6 pigs (3 barrows and 3 gilts) per pen. The control group (CON) were fed the diet that included 6% FM and 6% SPC supplement, and the 2 experimental groups were fed diets formulated by replacing all the FM (FMr) and SPC (SPCr) with DCP, respectively (Table 2). We envisage that DCP cannot completely replace both SPC and FM in actual condition, so the design allows it to replace only one of them. In this way, we can know which protein source can be replaced by DCP to provide better performance. The experiment period was divided into phase 1 (day 1–14 of post-weaning) and phase 2 (day 15–28 of post-weaning). All diets were formulated to meet or exceed the nutrient requirements recommended by NRC (2012), and 0.3% chromic oxide was added to the diet as the exogenous indicator.

### 2.2. Animal Management

All the piglets were fed in the experimental pens (1.2 m × 2 m) with 6 piglets per pen. Each pen was fitted with a duckbill drinker, an adjustable stainless-steel feeder and plastic slatted floors. Inside the pen, piglets were available to eat feed and drink water ad libitum. The pig house environment was controlled automatically, including the contents of CO_2_ and ammonium in the air, ventilation intensity, humidity and temperature. The average indoor temperature was controlled at 24–26 °C, while relative humidity was maintained at 60–70%. To prevent disease, the experimental house was cleaned every day and a health-assessment procedure was conducted by a veterinarian every week. After 12 h of starvation, the individual weight of each piglet and the remaining feed weight of each pen were weighed on day 0, 14 and 28, and then to calculate the average daily gain (ADG), average daily feed intake (ADFI) and the ratio of feed to gain (F:G). The incidence of diarrhea was calculated according to the following formula: diarrhea rate (%) = [(number of pigs with diarrhea × diarrhea days)/(number of pigs × total observed days)] × 100 [9]. The higher diarrhea rate represents more severe diarrhea in piglets.

### 2.3. Experimental Sample Collection

Before the experiment, 2 kg samples of DCP, FM and SPC were collected. During the experiment period, approximately 2 kg of representative feed samples were collected weekly. From day 12 to 14 and day 26 to 28, the rectal palpation was used every day to make sure approximately 100 g of fresh feces were collected from at least 3 medium BW pigs in a pen. All the fecal samples were frozen at −20 °C immediately after collection until analysis every day. Finally, the feces collected in the 3 days were pooled by pen and dried at 65 °C for 72 h. Before analysis, all these dried feces and feed samples were ground to pass through a 1 mm sieve.

On day 29, a total of 9 pigs with median BW in three groups (3 pigs each treatment) were humanely killed by exsanguination after electric shock. Segments of the mid-jejunum and mid-ileum were collected and rinsed with 0.9% saline, and then stored in 10% buffered formalin. The digesta collected from the cecum were frozen in liquid nitrogen and stored at −80 °C until further analysis.

### 2.4. Chemical Analysis

The dry matter (DM), crude protein (CP), ether extract (EE) and free gossypol of the ingredients (DCP, SPC and FM), diets and fecal samples were measured by following the methods of the Association of Official Agricultural Chemists [10]. The gross energy (GE) in the ingredients (DCP, SPC and FM), diets and fecal samples were measured by the automatic isoperibolic oxygen bomb calorimeter (Parr 1281, Automatic Energy Analyzer; Moline, IL, USA). Neutral detergent fiber (NDF) and acid detergent fiber (ADF) were determined by the procedure of Van Soest et al. [11]. Amino acids in DCP, SPC and FM were analyzed using ion-exchange chromatography with an automatic amino acid analyzer (L-8900, Automatic Amino Acid Analyzer; Hitachi, Tokyo, Japan) after hydrolyzing with 6 N HCl at 110 °C. Moreover, we used the atomic absorption spectrophotometer (Z-5000; Hitachi, Tokyo, Japan) to measure the concentration of chromic oxide (Cr) in feed and fecal samples. Organic matter (OM) was calculated from 1 − ash content (DM basis). Using the equation Apparent total tract digestibility nutrient (ATTD) = 1 − (Cr_diet_ × nutrient_feces_)/(Cr_feces_ × nutrient_diet_), nutrient digestibility was determined.

### 2.5. Measurement of Intestinal Morphology

Samples from the mid-jejunum and mid-ileum segments were embedded in paraffin and cut into 5-μm serial sections, and five non-successive sections from each tissue sample were selected and stained with hematoxylin-eosin for identification [12]. Six well-oriented villi (determined as the distance between the crypt openings and the end of the villi) and their associated crypt (measured from the crypt-villous junction to the base of the crypt) per section were selected and measured under a light microscope (CK-40, Olympus, Tokyo, Japan) at 40 × magnification and analyzed with an Image Analyzer (Lucia Software. Lucia, ZaDrahou, Czechoslovakia). The average of these measurements was calculated to yield a single value for each pig. These procedures were conducted by the same observer unaware of the dietary treatments.

### 2.6. Analysis of Microbial Community in Cecum

Until analysis, total genomic DNA was extracted from cecum digesta using a QIAamp DNA Stool Mini Kit (Qiagen, Germany) according to the manufacturer’s instructions. The final DNA concentration and purification were determined by NanoDrop 2000 UV-vis spectrophotometer (Thermo Scientific, Wilmington, DE, USA), and DNA quality was checked by 1% agarose gel electrophoresis. The bacteria 16S ribosomal RNA genes in the region of V3-V4 were amplified using polymerase chain reaction (PCR) with primers 338F (5′-ACTCCTACGGGAGGCAGCAG-3′) and 806R (5′-GGACTACHVGGGTWTCTAAT-3′). After extracting from a 2% agarose gel, the resulting PCR products were further purified using the AxyPrep DNA Gel Extraction Kit (Axygen Biosciences, Union City, CA, USA) and then quantified using QuantiFluor™-ST (Promega, Madison, Wisconsin, USA) according to the manufacturer’s protocol. Purified amplicons were pooled in equimolar and paired-end sequenced on an Illumina MiSeq platform (Illumina, San Diego, CA, USA) according to standard protocols by Majorbio Bio-Pharm Technology Co., Ltd. (Shanghai, China). Raw fastq files were quality-filtered by Trimmomatic and merged by FLASH. In addition, operational taxonomic units (OTUs) were defined as a similarity threshold of 0.97 using UPARSE. The taxonomy of each 16S rRNA gene sequence was analyzed by the RDP Classifier algorithm (http://rdp.cme.msu.edu/) [13] against the Silva (SSU123) 16S rRNA database using a confidence threshold of 70%.

### 2.7. Measurement of Volatile Acids and Branch-Chain Fatty Acids

The content of VFAs (acetate, propionate and butyrate) and BCFAs (isobutyrate, valerate and isovalerate) in the digesta were analyzed for evaluating the fermentation among three groups, based on the methods of He et al. [14]. The cecum digesta samples (0.4 g) were dissolved in 8 mL of ultrapure water, sonicated for 30 min, and then centrifuged at 3000× *g* for 5 min. Then, the collected supernatants were diluted (1:50) using ultrapure water and finally filtered into an injection vial through a 0.22 mm membrane. Each sample was detected using a high-performance ion chromatography system (DIONEX ICS-3000, Thermo Fisher, Waltham, MA, USA). The contents of VFAs and BCFAs were expressed as μmol/g of cecum digesta.

### 2.8. Statistical Analysis

Data for growth performance, diarrhea rate and digestibility of nutrients were analyzed with replicate as the experimental unit while intestinal morphology and fermentation were analyzed with pigs as the experimental unit. The data were subjected to ANOVA using the GLM procedure of SAS (version 9.4; SAS Inst. Inc., Cary, NC, USA). Statistical differences among treatments were separated by Student–Newman–Keul’s multiple range tests. Data for the bacterial community were analyzed with the three cecum samples per treatment. According to the guidance of R software, standardized OTUs reads were applied to analyze bacterial diversity by principal component analysis (PCA). In addition, the Kruskal–Wallis method was conducted to analyze the populations of the bacterial community in cecum samples of pigs at the phylum and family level. The abundance of bacteria at the phylum and family levels were shown as bar plots. Significant differences were defined at *p* < 0.05 and tendency was defined as 0.05 ≤ *p* < 0.10.

## 3. Results

### 3.1. Chemical Composition

As shown in Table 1, the NDF and ADF contents in DCP were greater than FM and SPC, but DM and GE in DCP were lower than those in FM and SPC. The CP content was similar in the three ingredients but differ in EE. Free gossypol is the unique anti-nutritional factor in DCP with a content of 224.6 mg/kg. The Arg, His and Met in DCP were greater but Ile, Leu and Lys were lower than FM and SPC. The concentrations of Thr, Try and Val in DCP was closer to the other two ingredients.

### 3.2. Growth Performance and Diarrhea Rate

As presented in Table 3, there were no significant differences in BW, ADG, ADFI and F:G in piglets among the three dietary groups in any period, except for ADFI in CON, which was greater than the other two groups in phase 2 (*p* < 0.01). Meanwhile, pigs fed the FMr diet showed a tendency for lower diarrhea frequency in phase 1 (d 1-d 14) (*p* = 0.06).

### 3.3. The Apparent Total Tract Digestibility of Nutrients

As shown in Table 4, from day 0 to day 14, greater apparent total tract digestibility (ATTD) of CP, GE, DM and OM were observed in the CON group compared with the FMr and SPCr groups (*p* < 0.01). In phase 2, there was no difference in the ATTD of nutrients among the three groups, and only ATTD of CP presented a decreased tendency in FMr compared with SPCr groups (*p* = 0.06).

### 3.4. Intestinal Morphology

The intestinal morphology was given in Table 5. The villus height of jejunum in the FMr and SPCr groups presented a decreased tendency compared with the CON group (*p* = 0.08). Meanwhile, the crypt depth of jejunum in the FMr group increased compared with the CON and SPCr groups (*p* < 0.01) but the ratio of villus height to crypt depth of jejunum in the FMr group was lower than it in the CON group (*p* = 0.03). No differences in the three indicators were founded in the ileum.

### 3.5. Cecum Microbiota

**Figure 1:**
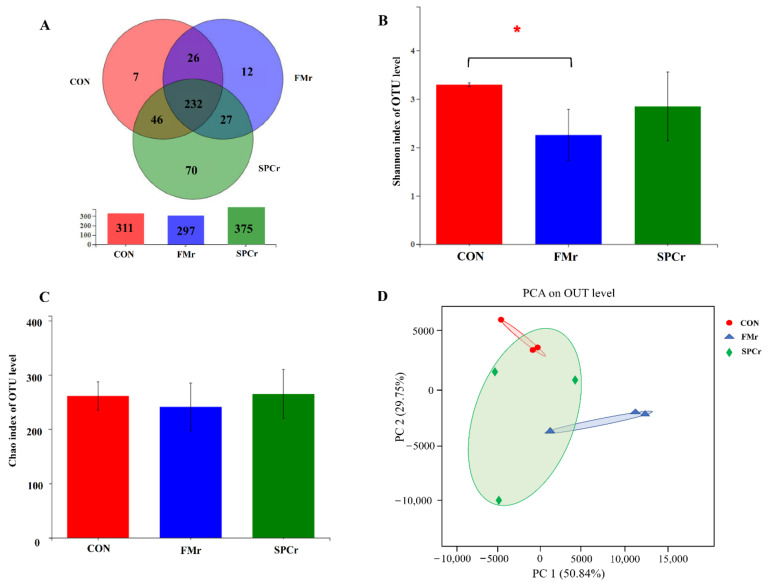
Richness, diversity, and similarity of bacterial communities between different dietary treatments in weaned pigs. Venn diagram of the OTUs in CON, FMr and SPCr groups (**A**). Bacterial diversity was estimated by the Shannon index (**B**). Bacterial richness was estimated by the Chao index (**C**). The principal component analysis (PCA) of samples in the bacterial community among the three groups (**D**). * represents a significant difference (*p* < 0.05).

The Venn analysis identified 311, 297 and 375 operational taxonomic unit (OTUs) from cecum samples in the CON, FMr and SPCr groups, respectively, and showed 7, 12 and 70 unique OUTs in the three groups (Figure 1A). The Shannon index in CON was higher than FMr group (Figure 1B) and no difference was observed in the Chao index (Figure 1C). The principal component analysis (PCA) showed that a greater variation was detected in cecum digesta samples of CON compared with the FMr group. The first two principal components accounted for 80.59% of the total variance in cecum microbiota composition (Figure 1D).

**Figure 2:**
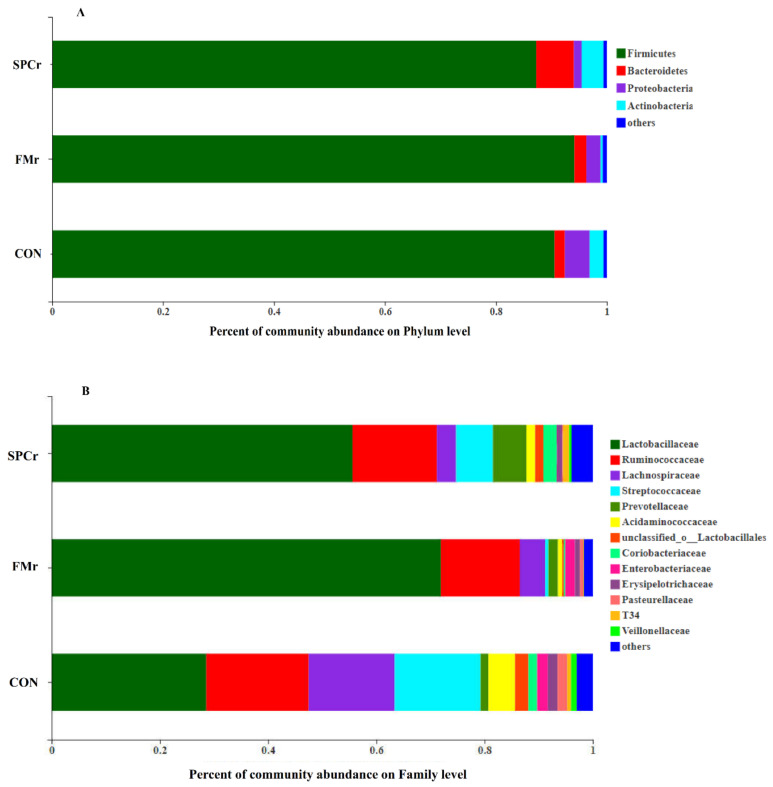
Community structures of cecum bacteria in CON, FMr, SPCr groups on phylum level (**A**) and family level (**B**). The bar plot was used to represent the difference of dominant species at the family levels by Kruskal–Wallis H test.

At the phylum level, Firmicutes, Bacteroidetes and Proteobacteria were the dominant bacteria, which accounted for more than 90% (Figure 2A) and no difference was determined (Table 6). Down to the family level, the predominant families with the Firmicutes phylum consisted of Lactobacillaceae, Streptococcaceae, Lachnospiraceae and Ruminococcaceae, while prevotellaceae, T34 were the predominate family in Proteobacteria and Bacteroidetes phylum, respectively (Figure 2B). The relative abundance of Lactobacillaceae in cecum digesta of FMr increased compared with CON while Ruminococcaceae, Lachnospiraceae and Streptococcaceae decreased (*p* < 0.05, Table 7).

### 3.6. Fermentation in Cecum

As shown in Table 8, replacing the FM with DCP decreased the concentrations of acetate in cecum compared with CON (*p* = 0.02). The FMr showed the greatest propionate and butyrate while the CON group contained the lowest butyrate in cecum digesta (*p* < 0.01). No differences were detected in total VFAs, isobutyrate, valerate and isovalerate in cecum digesta.

## 4. Discussion

### 4.1. Chemical Composition

The noticeable differences between the three ingredients (DCP, FM and SPC) lie in the fiber, EE, Lys, Met, Arg and free gossypol. Differing from fish meal, which is an animal protein source, SPC and DCP are plant-based protein ingredients that contain more plant cell walls and plant fibers, resulting in more ADF and NDF. However, SPC involves a stricter peeling process, which leads to a lower fiber concentration of SPC compared with DCP [15]. The higher fat content of fishmeal can be attributed to the high oil content of fish and the fact that both plant-derived proteins are subjected to an oil-extraction process. In the current study, DCP contained more Arg and Met and less Lys than FM and SPC; this finding was consistent with previous studies [1,8]. After the removal procedure, the free gossypol in DCP was significantly lower than that in CSM reported in previous literature with an average value of 300.94 mg/kg [16]. In addition, the tolerable level of free gossypol in growing–finishing pig diets was 100 mg/kg [17] and not reported in nursery pigs. The free gossypol level in diets in this study was lower than this tolerable level due to only 6% DCP being added to the diet, and no difference was observed in ADG in the three experimental diets.

### 4.2. Growth Performance and Nutrient Digestibility

In this experiment, replacing FM and SPC with DCP does not affect the growth performance of weaned pigs except for the ADFI in phase 2. The negative effect of a 6% replacement of DCP on ADFI can be attributed to the lower palatability compared with FM and SPC, which are always considered high-palatability ingredients [18]. Surprisingly, however, the difference in ADFI was only observed in phase 2 but not in phase 1. There is not a suitable explanation for this result, and it can only be speculated that prolonged exposure to the poorly palatable feed may cause a reduction in feed intake. In this study, we also found that using DCP decreased the ATTD of CP, GE, DM and OM in phase 1. This finding is consistent with Wang [8], who reported a decreased digestibility using a diet supplemented with DCP. Normally, animal protein is more digestible than plant protein due to the plant cell walls or fibers that may prevent the binding of digestive enzymes to nutrients. On the other hand, the higher fiber content in DCP may induce a rapid flow of digesta in the intestine and decrease the nutrient digestibility [19]. Meanwhile, free gossypol, the major anti-nutritional factor in cottonseed co-products [6], can react with free lysine and produce an indigestible complex, and finally result in lower protein digestibility [20]. The differences in ATTD of nutrients only appeared in phase 1 but not in phase 2 during the experiment period. Weaning usually changes the architecture and function of piglets’ gut [21], resulting in a temporary decrease in digestive and absorptive function of the small intestine. In phase 2, piglets are adapted to the diet and the intestine has developed maturely [22]. Therefore, the effect of different protein sources on the digestibility of pigs is reduced, which leads to no differences in digestibility in the later stage.

### 4.3. Intestinal Morphology

The intestinal morphology is a common marker to estimate the digestion and absorption capacity and the health of the intestine [23]. In this study, replacing FM with DCP would weaken the intestinal morphology in weaned pigs with a lower jejunum villus height and greater crypt depth. The free gossypol may be responsible for the morphological changes to the intestine since Li et al. (1990) [24] reported the hypersensitivity of the intestine to anti-nutritional factors in the diet. Marion et al. (2002) [25] also declared that 56% of the variation of villus height in the proximal small intestine was explained by the level of feed intake. With such a premise, the higher ADFI in CON in phase 2 can explain part of the intestinal morphology changes. This finding also supported the results of ATTD of nutrients due to the high correlation between intestinal morphology and nutrient absorption.

### 4.4. Cecum Microbiota

The gut microbiota offers many benefits to the host through a range of physiological functions such as strengthening gut integrity or shaping the intestinal epithelium [26]. The α-diversity is used as an indicator of functional resilience of the gut microbiota ecosystem, including species diversity (Shannon) and richness (Chao) [27]. In this study, the substitution of FM using DCP decreased the species diversity of cecum microbiota, but was not observed in the SPCr group. The three protein supplement ingredients were variable in the present study, which indicated that nutrition composition may be the main reason for microbiota changes. Polyphenols are the most common phytochemicals discovered in plants and can, directly and indirectly, affect the structure of gut microbiota by their antimicrobial and antioxidative properties [28]. The reduction of microbiota diversity when using DCP may be due to the bonding of polyphenols to the bacterial cell membrane and interrupting the normal bacterial functions. The DCP and SPC are both plant-origin proteins and contain more phytochemicals than FM, so the difference is only found in the FMr group. However, the data on the polyphenols of diets are non-accessible in this study, so this inference should be evaluated in further research. Additionally, fibers are poorly digested in the small intestine, but are an important substrate for hindgut microbiota, which then alters their composition. Therefore, an explanation for the shifts in the cecum when replacing FM with DCP may be an increasing fiber in the diet.

As with the previous report [29], Firmicutes and Bacteroidetes were found to be the dominating bacterial community at the phylum level in weaned pigs which count for approximately 95% of total gene sequences in cecum samples of weaned piglets. Our research did not detect any differences in bacterial abundance at the phylum level, but found changes in Lactobacillaceae Lachnospiraceae and Streptococcaceae at the family level.

In vitro, it has been proven that the dietary fiber of plants allows the selective proliferation of Lactobacillus strains [30]. As Lactobacillaceae become more dominant in cecum bacteria, the competition among the microbiota becomes more intense, resulting in a general decline in other microbiota. To be specific, the proliferation of Lactobacillaceae produces more lactate and brings down the pH, and the pH tolerance of other bacteria can also influence their composition [31]. Firmicutes were mainly composed of Lactobacillaceae, Streptococcaceae, Lachnospiraceae and Ruminococcaceae. The abundance of Streptococcaceae significantly decreased as Lactobacillaceae increased at the family level, which resulted in no difference in phylum level.

As mentioned before, the plant-origin ingredients usually contain more polyphenols, which can increase beneficial bacteria but reduce pathogenic bacteria [32]. Among these various bacteria, Lactobacillus is classified as a beneficial bacterium, which can produce hydrogen peroxide and antimicrobial factors (such as lactate and bacteriocins) to prevent the colonization of pathogenic bacteria [33]. Meanwhile, Lachnospiraceae is considered to be pathogenic bacteria because they produce potentially toxic metabolites that are harmful to the host. The decreased relative abundances of Lachnospiraceae and Ruminococcaceae might be beneficial for promoting growth or alleviating the incidence of diarrhea in weaning piglets [34]. In this study, there was no difference in the growth performance in the whole period. This may indicate that the improved cecum microbiota may relieve the negative effect of the ATTD of nutrients and weaken intestinal morphology.

### 4.5. Fermentation in Cecum

The cecum is the major site of microbial fermentation of undigested carbohydrates in pigs due to a great richness of bacteria and enough retention time of digesta [35]. Volatile fatty acids are the main microbial fermentation products in the gut, especially in the large intestine, and they are reported to have many beneficial effects on host health. In this research, replacing FM with DCP decreased the acetate and increased the butyrate and propionate. The microbial composition can change the VFA profile in the cecum [36]. The decreased Lachnospiraceae and Ruminococcaceae may explain the lower concentration of acetate because those two bacteria are known to produce acetate and suppress the growth of Bacteroidales [37]. Lactobacillus, Megasphaera, Blautia, and Prevotella are considered to participate in butyrate production [38]. Among them, Lactobacillus was thought to contact with butyrate production via expanded butyrate-producing bacterial strains such as Blautia, Roseburia, and Coprococcus [39]. The isobutyrate and isovalerate are only derived from the deamination of valine and leucine, respectively, which are often considered indicators of amino acid catabolism in the gut [40]. However, there were no differences in BCFAs, for two reasons. First, the amino acids in the diet have been balanced by supplying extra indispensable amino acids. Second, the digestion of protein or amino acids mostly takes place in the small intestine but not in the cecum. The significant differences in VFAs but not BCFAs also indicate that the effect on microbiota fermentation may be caused by carbohydrates and fibers but not by proteins. The result of the VFAs matches the shifts in cecum microbiota and prove that using DCP to replace FM can bring benefit changes in cecum microbiota.

## 5. Conclusions

A diet supplemented with 60 g/kg of DCP to replace FM and SPC weakens the intestinal morphology and decreases the nutrient digestibility, but improves the community structures of cecum microbiota, and may relieve these negative effects without impairing the growth performance of weaned piglets. Degossypolized cottonseed protein can be used as a cost-effective protein to replace FM and SPC.

## Figures and Tables

**Table 1:** The chemical compositions of degossypolized cottonseed protein, fish meal and soybean protein concentrate (as fed basis).

Items ^1^	DCP	FM	SPC
DM, %	90.43	93.84	93.14
GE, MJ/kg	18.24	18.67	19.27
CP, %	65.48	64.8	65.7
Ether extract, %	0.46	9.80	1.05
Neutral detergent fiber, %	20.42	-	8.60
Acid detergent fiber, %	5.51	-	5.44
Free gossypol, mg/kg	224.60	-	-
Indispensable amino acids			
Arginine, %	8.11	4.05	4.35
Histidine, %	2.17	1.18	1.68
Isoleucine, %	1.86	2.58	3.01
Leucine, %	3.71	4.73	5.04
Lysine, %	2.68	4.57	4.15
Methionine, %	3.30	1.69	0.88
Threonine, %	2.15	2.42	2.50
Tryptophan, %	0.79	0.86	0.81
Valine, %	2.87	2.85	3.17

^1^ DCP, degossypolized cottonseed protein; FM, fish meal; SPC, soy protein concentrate; DM, dry matter; DE digestible energy; ME, metabolic energy; CP, crude protein.

**Table 2:** The ingredient composition and nutrient levels of diets (as fed basis).

Items ^1^, %	CON	FMr	SPCr
Corn	53.67	52.68	53.53
Soybean meal	16	16	16
SPC	6	6	0
FM	6	0	6
DCP	0	6	6
Whey powder	10	10	10
Soybean oil	2.9	2.9	2.9
Sucrose	2	2	2
Limestone	0.7	1	0.7
Dicalcium phosphate	0.92	1.4	0.9
Salt	0.25	0.25	0.25
L-Lysine·HCl	0.47	0.62	0.6
L-Threonine	0.17	0.19	0.19
L-Tryptophan	0.03	0.03	0.04
DL-Methionine	0.09	0.13	0.09
Chromic oxide	0.3	0.3	0.3
Premix	0.5	0.5	0.5
Calculated nutrient level			
ME, kcal/kg	3466	3455	3466
Crude protein	20.81	21.09	21.02
SID Lys	1.36	1.36	1.36
SID Thr	0.8	0.8	0.8
SID Met	0.4	0.4	0.4
SID Trp	0.22	0.22	0.22
Ca	0.81	0.8	0.8
Digestible phosphorus	0.41	0.4	0.4
Measured nutrient level			
GE, kcal/kg	4005	3951	3975
Crude protein	19.56	19.8	19.78
Ether extract	5.61	5.01	5.56
Neutral detergent fiber	9.28	10.28	9.56
Acid detergent fiber	2.61	3.11	2.74

SID, standardized ileal digestibility; DCP, degossypolized cottonseed protein; FM, fish meal; SPC, soybean protein concentrate; ME, metabolic energy. ^1^ Remix provided the following quantities per kilogram of complete diet for weaned piglets: vitamin A, 12,000 IU; vitamin D_3_, 3000 IU; vitamin E, 30 IU; vitamin K_3_, 2.5 mg; vitamin B_12_, 20 μg; riboflavin, 4.0 mg; pantothenic acid, 12.5 mg; niacin, 40 mg; choline chloride, 400 mg; folacin, 0.7 mg; thiamine 2.5 mg; pyridoxine 3.0 mg; biotin, 70 μg; Mn, 30 mg (MnO); Fe, 100 mg (FeSO_4_·H_2_O); Zn, 80 mg (ZnO); Cu, 90 mg (CuSO_4_·5H_2_O); I, 0.25 mg (KI); Se, 0.15 mg (Na_2_SeO_3_).

**Table 3:** Effects of replacing fish meal and soybean protein concentrate with degossypolized cottonseed protein on growth performance and diarrhea rate in weaning pigs.

Item ^1^	CON	FMr	SPCr	SEM	*p*-Value
Initial BW, kg	8.04	8.06	8.07	0.26	0.96
Day 14 BW, kg	12.96	13.34	13.33	0.44	0.75
Day 28 BW, kg	21.81	22.05	21.65	0.71	0.70
Day 1 to 14					
ADG, g/d	351	377	376	17.21	0.50
ADFI, g/d	479	490	510	19.91	0.55
F:G	1.41	1.29	1.32	0.07	0.58
diarrhea rate, %	4.64	3.93	7.26	0.87	0.06
Day 15 to 28					
ADG, g/d	632	622	594	26.88	0.61
ADFI, g/d	962^a^	892^b^	874^b^	15.76	< 0.01
F:G	1.54	1.43	1.47	0.06	0.43
diarrhea rate, %	0.72	1.67	1.19	0.28	0.11
Day 1 to 28					
ADG, g/d	492	500	485	17.67	0.85
ADFI, g/d	720	684	689	12.02	0.13
F:G	1.49	1.36	1.42	0.05	0.25
diarrhea rate, %	2.68	2.80	4.23	0.53	0.13

ADFI, average daily feed intake; ADG, average daily gain; BW, body weight; F:G, feed to gain ratio; DCP, degossypolized cottonseed protein; SPC, soy protein concentrate; FM, fish meal. ^1^ FMr: 6% FM in CON were all replaced with DCP; SPCr: 6% SPC in CON were all replaced with DCP.

**Table 4:** Effects of replacing fish meal and soybean protein concentrate with degossypolized cottonseed protein on apparent total tract digestibility in weaning pigs.

Items ^1^	CON	FMr	SPCr	SEM	*p*-Value
Day 14					
CP, %	74.33 ^a^	69.46 ^b^	69.32 ^b^	0.95	<0.01
GE, %	82.17 ^a^	78.3 ^b^	77.19 ^b^	0.71	<0.01
DM, %	81.63 ^a^	77.35 ^b^	76.78 ^b^	0.72	<0.01
OM, %	84.67 ^a^	81.21 ^b^	80.51 ^b^	0.60	<0.01
Day 28					
CP, %	72.9	70.57	73.79	0.83	0.06
GE, %	80.18	78.67	80.03	0.72	0.32
DM, %	80.03	78.65	78.78	0.67	0.33
OM, %	82.96	82.09	82.27	0.51	0.49

CP, crude protein; GE, gross energy; DM, dry matter; OM, Organic matter. SPC, soy protein concentrate; FM, fish meal. ^1^ Mean values within a row with different letters differ at *p* < 0.05.

**Table 5:** Effects of experimental diets on intestinal morphology in weaned pigs.

Item ^1^	CON	FMr	SPCr	SEM	*p*-Value
Jejunum					
Villus height, μm	419	399	393	5.99	0.08
Crypt depth, μm	198 ^b^	224 ^a^	202 ^b^	3.64	<0.01
Villus height:crypt depth	2.13 ^a^	1.78 ^b^	1.96 ^ab^	0.05	0.03
Ileum					
Villus height, μm	346	326	336	8.78	0.35
Crypt depth, μm	161	161	160	6.73	0.98
Villus height:crypt depth	2.15	2.02	2.12	0.12	0.75

^1^ Mean values within a row with different letters differ at *p* < 0.05.

**Table 6:** Differences in the cecum digesta microbiota composition at phylum levels.

Items ^1^	CON	FMr	SPCr	*p*-Value
Firmicutes, %	90.53 ± 1.71	95.26 ± 2.54	86.13 ± 9.24	0.18
Bacteroidetes, %	1.86 ± 0.89	2.30 ± 1.45	6.64 ± 8.51	0.88
Proteobacteria, %	4.48 ± 3.55	0.72 ± 0.99	3.24 ± 3.58	0.30
Actinobacteria, %	2.51 ± 1.63	0.99 ± 0.99	3.31 ± 2.79	0.43

^1^ The values were presented as means ± SD. Only bacteria with a concentration greater than 1% were given.

**Table 7:** Differences in the cecum digesta microbiota composition at family levels.

Items ^1, 2^	CON	FMr	SPCr	*p*-Value
Lactobacillaceae, %	28.56 ^b^ ± 7.78	83.74 ^a^ ± 1.14	43.75 ^ab^ ± 16.09	0.04
Ruminococcaceae, %	18.93 ± 6.27	5.43 ± 1.58	24.75 ± 15.29	0.06
Lachnospiraceae, %	15.91 ^a^ ± 8.14	3.32 ^b^ ± 1.49	4.91 ^b^ ± 1.20	0.04
Streptococcaceae, %	15.85 ^a^ ± 6.00	0.41 ^b^ ± 0.42	7.02 ^ab^ ± 7.08	0.04
Prevotellaceae, %	1.48 ± 1.88	1.88 ± 1.21	6.09 ± 7.99	0.96
Acidaminococcaceae, %	4.89 ± 0.97	0.97 ± 0.66	1.38 ± 1.33	0.96
unclassified_o_Lactobacillales, %	2.48 ± 0.22	0.22 ± 0.23	1.67 ± 2.24	0.20
Coriobacteriaceae, %	1.63 ± 1.13	0.67 ± 0.69	2.07 ± 1.67	0.43
Enterobacteriaceae, %	1.96 ± 3.30	0.01 ± 0.02	1.80 ± 3.08	0.25
Erysipelotrichaceae, %	1.83 ± 0.57	0.32 ± 0.02	1.56 ± 0.90	0.06
Pasteurellaceae, %	1.77 ± 0.01	0.01 ± 0.01	0.81 ± 0.99	0.06
T34, %	0.71 ± 1.22	0.66 ± 0.99	0.50 ± 0.44	0.83
Veillonellaceae, %	1.06 ± 1.64	0.10 ± 0.11	0.38 ± 0.36	0.56

^1^ The values were presented as means ± SD. Only bacteria with a concentration greater than 0.50% were given. ^2^ Mean values within a row with different letters differ at *p* < 0.05.

**Table 8:** Effects of replacing fishmeal and soybean protein concentrate with degossypolized cottonseed protein on concentrations of volatile fatty acids and branch-chain fatty acids in the cecum digesta (μmol/g).

Items ^1^	CON	FMr	SPCr	SEM	*p*-Value
VFAs					
Acetate	95.913 ^a^	88.601 ^b^	92.326 ^ab^	1.273	0.02
Propionate	61.080 ^b^	66.377 ^a^	64.940 ^ab^	0.940	0.02
Butyrate	11.166 ^c^	13.726 ^a^	12.523 ^b^	0.170	<0.01
Total VFAs	168.159	168.704	169.789	0.159	0.89
BCFAs					
Isobutyrate	2.301	2.101	2.35	0.072	0.11
Valerate	5.015	5.023	5.155	0.109	0.62
Isovalerate	3.600	3.425	3.556	0.070	0.26

VFAs: volatile fatty acids; BCFAs: branch-chain fatty acids. ^1^ Mean values within a row with different letters differ at *p* < 0.05.

## Data Availability

The data presented in this study are available from the corresponding author on request.
